# Obesity with metabolic abnormality is associated with the presence of carotid atherosclerosis in Korean men: a cross-sectional study

**DOI:** 10.1186/s13098-015-0063-y

**Published:** 2015-08-19

**Authors:** Ha-Na Kim, Se-Hong Kim, Young-Mi Eun, Sang-Wook Song

**Affiliations:** Department of Family Medicine, College of Medicine, St. Vincent’s Hospital, The Catholic University of Korea, Seoul, Republic of Korea

**Keywords:** Obesity, Metabolic syndrome, Carotid atherosclerosis, Carotid plaque, Carotid intima-media thickness

## Abstract

**Background:**

Obesity is a risk factor for cardiovascular disease, but metabolic disturbances can also lead to the development of this disease. Therefore, we investigated the associations between obesity subtype, considering both body weight and metabolic disturbances, and carotid atherosclerosis as a predictor of cardiovascular disease in Korean men.

**Methods:**

Data from a total of 980 men were analysed in this study. Obesity subtypes were classified as normal weight without metabolic syndrome (metabolically healthy normal weight; MHNW), obesity without metabolic syndrome (metabolically healthy, but obese; MHO), normal weight with metabolic syndrome (metabolically abnormal, but normal weight; MANW) and obesity with metabolic syndrome (metabolically abnormal obese; MAO). Carotid intima-media thickness (CIMT) and carotid plaque were assessed using a high-resolution B-mode ultrasound system. Carotid atherosclerosis was defined as a mean CIMT value >0.9 mm or the presence of carotid plaque.

**Results:**

Mean CIMT in the MAO subtype was significantly higher than that in the MHNW control group (0.790 ± 0.019 vs. 0.747 ± 0.013 mm; p < 0.001). The presence of carotid plaque was positively associated with MAO subtype [adjusted odds ratio (aOR) 1.49, 95 % confidence interval (CI) 1.02–2.16; p = 0.039], but not with MHO or MANW, compared to the MHNW control group. The MAO subtype showed a positive association with the presence of carotid atherosclerosis (aOR 1.68, 95 % CI 1.17–2.42; p = 0.006).

**Conclusions:**

Only the MAO subtype showed a higher CIMT value and positive associations with carotid plaque and carotid atherosclerosis, but not with MHO and MANW subtypes, compared to the MHNW control. Additional prospective studies are needed to evaluate preclinical carotid atherosclerosis according to the subtypes of obesity.

## Background

Obesity, which is defined as excess accumulation of body fat, is a major public health problem with a prevalence that is increasing worldwide [1]. Obesity is associated with an increased risk of cardiometabolic diseases, such as cardiovascular disease (CVD) and stroke. In addition, obesity-induced insulin resistance and metabolic abnormalities also cause cardiometabolic disease [2]. However, not all obese individuals show metabolic abnormalities, and not all normal-weight individuals show favourable metabolic conditions. Therefore, obesity subtypes divided according to metabolic status such as metabolically healthy but obese (MHO), metabolically abnormal but normal weight (MANW), and metabolically abnormal obese (MAO) have attracted attention [3, 4].

Several epidemiological studies have reported CVD/stroke risks according to obesity subtypes, but the results have been inconsistent. In a prospective cohort study, only MAO subtype showed increased rates of CVD/stroke events compared to metabolically healthy normal weight (MHNW) [5], whereas CVD risks in MANW and MAO subtypes, which include participants with metabolic disturbances regardless of body weight, were higher but, MHO subtype showed similar CVD risks than those in MHNW controls [6]. Furthermore, there have been few studies regarding the associations between obesity subtypes and CVD/stroke risk in Asia [7, 8].

Measurement of carotid intima-media thickness (CIMT) is a simple and non-invasive imaging tool for assessing structure and change in the carotid arterial wall. Increased CIMT is widely considered an indicator of atherosclerosis [9], and is linked to various adverse cardiovascular outcomes [10, 11]. Furthermore, carotid plaque is also associated with an increased risk of CVD/stroke events [12, 13], and appears to be a more powerful predictor of CVD/stroke risk than CIMT alone [14]. Therefore, measurement of CIMT levels along with the assessment of carotid plaque is one of the important methods in the cardiovascular risk prediction. To date, however, few studies have addressed the association between the subtypes of obesity, CIMT levels, and carotid plaques [15–17]. Therefore, we investigated whether various obesity subtypes are associated with CIMT values, the presence and severity of carotid plaques, and carotid atherosclerosis in Korean men.

## Methods

### Study population

The initial study population consisted of 1043 men who visited the health promotion centre of St. Vincent’s Hospital, Catholic University of Korea, Suwon, South Korea, for a health check-up between January 2011 and December 2014. We excluded subjects with a history of previous CVD/stroke events (*n* = 33), or with thyroid disease (*n* = 8), cancer (*n* = 19), or decreased kidney function [estimated glomerular filtration rate (eGFR) <30 mL/min/1.73 m^2^; *n* = 3]. In total, 980 men were included in the study. The study protocol was approved by the Catholic University of Korea, St. Vincent’s Hospital, Institutional Review Board (IRB approval number: VC15RISI0072).

### Classification of obesity

BMI level was classified as normal weight or obesity according to a BMI <25 kg/m^2^ and ≥25.0 kg/m^2^, respectively [18]. The metabolically abnormal status was defined as having any three or more of the following, consistent with the revised National Cholesterol Education Program Adult Treatment Panel III (NCEP-ATP III) to define metabolic syndrome (MetS) [19]: waist circumference ≥90 cm [20]; triglyceride level ≥150 mg/dL or taking medication for increased triglycerides; high-density lipoprotein cholesterol (HDL-C) level <40 mg/dL or taking medication to improve HDL-C; systolic blood pressure ≥130 mmHg or diastolic blood pressure ≥85 mmHg or taking anti-hypertensive agent; fasting glucose level ≥100 mg/dL or taking blood glucose-lowering agent. The subtypes of obesity were divided into four groups based on the presence/absence of obesity and MetS: MHNW, MHO, MANW, and MAO.

### Assessment of CIMT and carotid plaque

Assessment of the carotid arteries was measured with a high-resolution B-mode ultrasound system (ACUSON Antares; Siemens, Erlangen, Germany) with a linear 9–13 MHz transducer, recorded by a single trained technician, and examined once per subject. CIMT, which is the distance between the luminal-intimal interface and the medial-adventitial interface, was measured by conventional eye-measurement method at each of three levels on the far walls of the right and left common carotid arteries, 1.5 cm proximal to the bifurcation, avoiding the sites of plaque. The average of maximal values of the right and left CIMT were used as a mean CIMT value. An increased mean CIMT was defined as mean CIMT value >0.9 mm [21]. For assessment of carotid plaque, the far and near walls of the right and left common carotid arteries, bulbs, and internal carotid arteries were scanned. Carotid plaque, as a focal thickening encroaching into the lumen by 0.5 mm or by 50 % of the surrounding CIMT or where the intima-media thickness is ≥1.5 mm [21], was classified according to surface characteristics, echogenicity, and texture. Surface characteristics were classified as smooth, mildly irregular (height variation in ≤0.4 mm), markedly irregular (height variation in >0.4 mm), or ulcerated (discrete depressions of >2 mm in width extending into the media). Echogenicity was characterised as hypoechoic, isoechoic, hyperechoic, or calcified. Texture was classified as homogeneous or heterogeneous. In cases with multiple focal lesions, the largest lesion was assessed. The severity of carotid plaque was classified as no plaque, moderate-risk plaque (remaining plaques, not including high-risk plaques), and high-risk plaque (the presence of markedly irregular or ulcerated surface, or hypo-echogenicity, or heterogeneous texture on plaques occupying >50 % of the total plaque volume). In the present study, carotid atherosclerosis was defined by the increased mean CIMT or the presence of carotid plaque [21].

### Clinical and anthropometric measurements

Anthropometric measurements were obtained by specially trained examiners. Height and weight were measured after an overnight fast while the participants wore a lightweight gown, and waist circumference was measured using a measuring tape in the horizontal plane around the umbilical region after exhaling. Blood pressure measurements were taken in the sitting position after a rest period of at least 5 min. BMI was calculated as each participant’s weight in kilograms divided by the square of their height in meters.

Self-reported information regarding age, smoking, alcohol consumption, amount of physical activity, and medical histories were obtained. Subjects were divided into three categories based on current cigarette use estimates: non-smoker, ex-smoker, or current smoker. Subjects were divided into three categories based on alcohol consumption: abstinence (no alcoholic drinks consumed within the last year), moderate drinking (less than 14 standard drinks consumed per week), and heavy drinking (more than 14 standard drinks consumed per week). Low physical activity was defined as 150 min or less of moderate intensity or 75 min or less of vigorous intensity exercise per week. Past histories of hypertension, dyslipidemia, or diabetes mellitus were defined as a previous diagnosis with high blood pressure, dyslipidemia, or diabetes before this examination, and use of anti-hypertensive, lipid, or blood glucose-lowering agents.

### Laboratory measurements

Blood samples to measure biochemical parameters were collected from the antecubital vein of each participant after an overnight fast. Glucose, total cholesterol, triglyceride, low-density lipoprotein cholesterol (LDL-C), HDL-C, and creatinine levels were determined using an auto-analyser (Hitachi 747; Hitachi, Tokyo, Japan), and fasting plasma insulin levels were determined by radioimmunoassay (Immulite 2000 XPi system; Siemens, Erlangen, Germany). Insulin resistance was assessed using the homeostasis model assessment insulin resistance (HOMA-IR) index, which was calculated as follows: [fasting glucose (mg/dL) × fasting insulin (μIU/mL)]/405 [22]. The glomerular filtration rate was estimated by the re-expressed Modification of Diet in Renal Disease study equation using calibrated serum creatinine values [23]; the formula used for eGFR was as follows: 175 × (serum creatinine concentration)^−1.154^ × (age)^−0.203^.

### Statistical analysis

The characteristics of the study participants were analysed by one-way analysis of variance for continuous variables and the Chi square test for dichotomous variables. The data are expressed as mean ± standard deviations or percentages and as medians with interquartile ranges for skewed distributions. The variables with skewed distributions were analysed after logarithmic transformation. Correlation and regression coefficients between mean CIMT and associated risk factors were obtained by simple and multiple linear regression analyses. The differences in mean values of CIMT according to the subtypes of obesity were evaluated by analysis of covariance with age, smoking, alcohol consumption, physical activity, total cholesterol, LDL-C, HOMA-IR index, and eGFR levels as covariates. We also examined the relationships between the presence and severity of plaque, or the presence of carotid atherosclerosis as dependent variables and subtypes of obesity as the independent variables, using multiple logistic regression analysis. Model 1 was adjusted for age, model 2 was adjusted for age, smoking, alcohol consumption, and physical activity, and model 3 was adjusted for age, smoking, alcohol consumption, physical activity, total cholesterol, LDL-C, HOMA-IR index, and eGFR levels. The percentages of participants with or without MetS regarding the presence/absence of obesity were analysed using the Chi square test. With regard to obesity, the associations between increased mean CIMT, the presence of carotid plaque or carotid atherosclerosis and the presence of MetS were examined by multiple logistic regression analysis adjusted using the abovementioned variables as covariates. The statistical analyses were performed using SPSS software for windows (ver. 21.0, IBM Corp., Armonk, NY, USA). In all of the analyses, p < 0.05 was taken to indicate statistical significance.

## Results

In a total of 980 men aged 19–80 years of age (mean age, 49.9 years), the prevalence rates of MHNW, MHO, MANW, and MAO types were 41.4, 24.6, 8.2, and 25.8 %, respectively. Significant differences in age, past medical history, waist circumference, systolic and diastolic blood pressures, fasting glucose, triglycerides, HDL-C, HOMA-IR index, and eGFR levels as well as mean CIMT and the presence of carotid plaque were observed according to the subtypes of obesity (Table 1).

**Table 1:** Characteristics of the study participants according to subtypes of obesity

	All	MHNW	MHO	MANW	MAO	p value
N (%)	980 (100.0)	406 (41.4)	241 (24.6)	80 (8.2)	253 (25.8)	–
Age (years)	49.9 ± 10.8	49.8 ± 11.1	47.3 ± 10.7	55.2 ± 10.2	50.7 ± 9.9	<0.001
Current smoking (%)	36.1	36.2	37.2	28.2	37.2	0.378
Heavy drinking (%)	28.1	26.1	31.5	28.6	27.9	0.542
Low physical activity (%)	59.1	57.1	56.1	70.0	61.8	0.273
Diabetes mellitus (%)	11.6	7.2	4.2	31.3	19.4	<0.001
Hypertension (%)	24.5	13.8	16.3	43.8	43.1	<0.001
Dyslipidemia (%)	4.0	2.2	4.2	3.8	6.7	0.041
Fasting glucose (mg/dL)^a^	95 (88–105)	91 (85–98)	92 (87–98)	105 (100–139)	104 (94–119)	<0.001
Total cholesterol (mg/dL)^a^	199 (174–223)	199 (176–223)	201 (176–226)	194 (178–221)	205 (172–231)	0.767
Triglycerides (mg/dL)^a^	130 (92–184)	102 (75–140)	113 (90–146)	183 (141–235)	177 (135–242)	<0.001
HDL-C (mg/dL)^a^	43 (37–47)	47 (42–55)	44 (40–49)	37 (34–42)	37 (34–44)	<0.001
LDL-C (mg/dL)	122.8 ± 32.1	123.1 ± 30.6	124.8 ± 32.8	121.2 ± 36.7	121.2 ± 32.3	0.613
HOMA-IR index^a^	0.84 (0.46–1.75)	0.51 (0.42–1.05)	0.86 (0.47–1.55)	1.29 (0.63–2.14)	1.59 (0.82–2.69)	<0.001
eGFR (mL/min/1.73 m^2^)	92.3 ± 16.1	94.5 ± 14.3	91.7 ± 17.2	89.3 ± 17.4	90.2 ± 16.9	0.002
SBP (mmHg)	127.0 ± 12.8	123.2 ± 12.9	124.7 ± 12.4	134.0 ± 10.6	131.6 ± 11.5	<0.001
DBP (mmHg)	77.4 ± 9.5	75.1 ± 9.3	75.5 ± 9.5	81.9 ± 8.6	80.5 ± 9.0	<0.001
Waist circumference (cm)	88.0 ± 7.4	82.2 ± 5.1	91.2 ± 4.4	85.8 ± 4.1	95.1 ± 5.7	<0.001
Body mass index (kg/m^2^)	25.1 ± 2.9	22.6 ± 1.7	26.8 ± 1.7	23.5 ± 1.1	27.9 ± 2.2	<0.001
Mean CIMT (mm)^a^	0.75 (0.65–0.88)	0.72 (0.62–0.84)	0.72 (0.62–0.84)	0.77 (0.67–0.91)	0.80 (0.70–0.90)	<0.001
Carotid plaque (%)	28.1	26.6	22.4	33.8	34.0	0.019
Carotid atherosclerosis (%)	38.7	36.0	33.2	46.3	45.8	0.008

*CIMT* carotid intima-media thickness, *DBP* diastolic blood pressure, *eGFR* estimated glomerular filtration rate, *HDL-C* high-density lipoprotein cholesterol, *HOMA-IR* homeostasis model assessment insulin resistance, *LDL-C* low-density lipoprotein cholesterol, *MANW* metabolically abnormal, but normal weight, *MAO* metabolically abnormal obese, *MHO* metabolically healthy, but obese, *MHNW* metabolically healthy normal weight, *SBP* systolic blood pressure

^a^Values are expressed as means ± standard deviation or percentages and as medians (interquartile ranges) for skewed distribution

As shown in Table 2, significant correlations were observed between age, heavy drinking, past histories of diabetes or hypertension, BMI, LDL-C, HOMA-IR index, eGFR, the presence of MetS, and mean CIMT by simple regression analysis (p < 0.05). Stepwise multiple regression analysis indicated that age, current smoking, diabetes, BMI, and LDL-C values were positive independent determinants of mean CIMT (p < 0.005), whereas the presence of MetS was not.

**Table 2:** Correlations between carotid intima-media thickness value and associated risk factors

	Correlations with CIMT value			
	r	p value	ß	p value
Age (years)	*0.555*	*<0.001*	*0.012*	*<0.001*
Current smoking	−0.003	0.933	*0.025*	*0.004*
Heavy drinking	*0.087*	*0.007*	–	–
Low physical activity	0.015	0.648	–	–
Diabetes mellitus	*0.210*	*<0.001*	*0.097*	*<0.001*
Hypertension	*0.153*	*<0.001*	–	–
Dyslipidemia	0.044	0.168	–	–
SBP (mmHg)	0.077	0.050	–	–
DBP (mmHg)	−0.006	0.887	–	–
Body mass index (kg/m^2^)	*0.088*	*0.006*	*0.011*	*<0.001*
Total cholesterol (mg/dL)^a^	0.044	0.173	–	–
LDL-C (mg/dL)	*0.083*	*0.010*	*0.001*	*<0.001*
HOMA-IR index^a^	*0.138*	*<0.001*	–	–
eGFR (mL/min/1.73 m^2^)	−*0.190*	*<0.001*	–	–
Metabolic syndrome	*0.180*	*<0.001*	–	–

Correlation coefficient (r) and regression coefficient (ß) were obtained by simple- and multiple linear regression analysis. Results in italics indicate statistical significance at the 0.05 level

*CIMT* carotid intima-media thickness, *DBP* diastolic blood pressure, *eGFR* estimated glomerular filtration rate, *HOMA-IR* homeostasis model assessment insulin resistance, *LDL-C* low-density lipoprotein cholesterol, *SBP* systolic blood pressure

^a^Variables with skewed distributions performed log-transformation

**Fig. 1:**
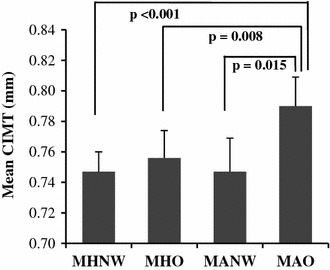
Carotid intima-media thickness according to obesity subtypes. Data are expressed as mean ± standard deviation. Only significant p values in CIMT differences among groups are presented. *CIMT* carotid intima-media thickness, *MHNW* metabolically healthy normal weight, *MHO* metabolically healthy, but obese, *MANW* metabolically abnormal, but normal weight, *MAO* metabolically abnormal obese

The mean CIMT values adjusted for covariates, such as age, smoking, alcohol consumption, physical activity, total cholesterol, LDL-C, HOMA-IR index, and eGFR levels according to subtypes of obesity are shown in Fig. 1. Mean CIMT increased according to group in the order MHO, MANW, and MAO compared to MHNW controls (p for trend <0.001). Mean CIMT of MHNW control, MHO, MANW, and MAO subtypes were 0.747 ± 0.013, 0.752 ± 0.018, 0.757 ± 0.030, and 0.790 ± 0.019 mm, respectively. Mean CIMT in MAO was significantly higher than that in the MHNW group (p < 0.001), but no differences in mean CIMT values were seen among the MHNW control, MHO and MANW subtypes.

After adjusting for age (model 1), the adjusted odds ratio (aOR) of the presence of carotid plaque in MAO was 1.49 [95 % confidence interval (CI) 1.01–2.00; p = 0.043], and the presence of carotid plaque was still positively associated with MAO subtype after adjusting for age, smoking, alcohol consumption, and physical activity (model 2) (aOR 1.49, 95 % CI 1.03–2.16; p = 0.036). The positive association between MAO subtype and the presence of carotid plaque remained significant (aOR 1.49, 95 % CI 1.02–2.16; p = 0.039) after adjusting for age, smoking, alcohol consumption, physical activity, total cholesterol, LDL-cholesterol, HOMA-IR index, and eGFR levels (model 3). The presence of moderate-risk plaque was associated with MAO subtype after adjusting for age, smoking, alcohol consumption, physical activity (model 2) (aOR 1.65, 95 % CI 1.01–2.68, p = 0.046), and the association remained significant after adjusting for the abovementioned covariates (model 3) (aOR 1.87, 95 % CI 1.11–3.14; p = 0.019), but the presence of high-risk plaque was not associated with subtypes of obesity. No significant association was found between the presence or severity of carotid plaques and MHO or MANW subtypes (Table 3).

**Table 3:** Odds ratios and 95 % confidence intervals for carotid plaque according to subtypes of obesity

	MHNW	MHO	MANW	MAO
Presence of plaque				
Crude	1	0.80 (0.55–1.16)	1.41 (0.84–2.35)	1.43 (0.99–2.06)
Model 1	1	0.98 (0.65–1.46)	0.96 (0.55–1.68)	*1.42* (*1.01–2.00*)
Model 2	1	0.98 (0.65–1.47)	0.91 (0.51–1.61)	*1.49* (*1.03*–*2.16*)
Model 3	1	0.98 (0.64–1.48)	0.95 (0.53–1.70)	*1.49* (*1.02*–*2.16*)
Plaque, moderate risk				
Prevalence (%)	10.8	12.0	15.0	15.4
Crude	1	1.05 (0.63–1.73)	1.53 (0.76–3.09)	1.58 (0.99–2.53)
Model 1	1	1.22 (0.72–2.04)	1.12 (0.54–2.31)	1.57 (0.97–2.54)
Model 2	1	1.16 (0.68–1.97)	1.09 (0.51–2.33)	*1.65* (*1.01*–*2.68*)
Model 3	1	1.28 (0.74–2.20)	1.11 (0.50–2.44)	*1.87* (*1.11*–*3.14*)
Plaque, high risk				
Prevalence (%)	15.8	10.4	18.8	18.6
Crude	1	0.62 (0.38–1.02)	1.32 (0.69–2.49)	1.31 (0.86–1.99)
Model 1	1	0.77 (0.45–1.30)	0.84 (0.42–1.67)	1.33 (0.84–2.09)
Model 2	1	0.78 (0.45–1.34)	0.89 (0.44–1.80)	1.39 (0.87–2.19)
Model 3	1	0.84 (0.48–1.45)	0.71 (0.33–1.50)	1.25 (0.76–2.07)

Values are expressed as odds ratio (95 % confidence interval). Model 1 adjustment for age, Model 2 adjustment for Model 1 + smoking, alcohol consumption, physical activity, Model 3 Model 2 + total cholesterol, LDL-C, HOMA-IR index and eGFR levels. Results in italics indicate statistical significance at the 0.05 level

*MANW* metabolically abnormal, but normal weight, *MAO* metabolically abnormal obese, *MHO* metabolically healthy, but obese, *MHNW* metabolically healthy normal weight

**Fig. 2:**
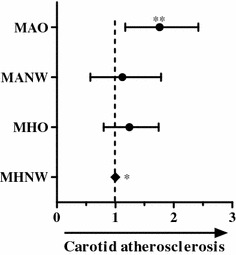
Adjusted odds ratios and 95 % confidence intervals of carotid atherosclerosis according to obesity subtypes. *Reference, **p value <0.05. *MHNW* metabolically healthy normal weight, *MHO* metabolically healthy, but obese, *MANW* metabolically abnormal, but normal weight, *MAO* metabolically abnormal obese

After adjusting for age, smoking, alcohol consumption, physical activity, past medical history, total cholesterol, LDL-C, HOMA-IR index, and eGFR levels, the aORs of carotid atherosclerosis in the MHO, MANW, and MAO subtypes were 1.18 (95 % CI 0.80–1.74; p = 0.294), 1.01 (95 % CI 0.57–1.78; p = 0.575), and 1.68 (95 % CI 1.17–2.42; p = 0.006), respectively, compared to MHNW controls; thus, only the MAO subtype was positively associated with the presence of carotid atherosclerosis (Fig. 2).

**Fig. 3:**
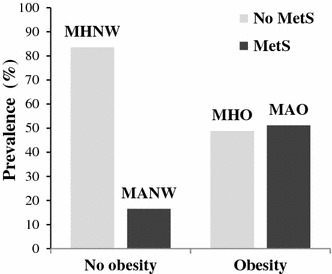
Prevalence of metabolic syndrome according to obesity. Obesity was defined as a body mass index ≥25.0 kg/m^2^. *MetS* metabolic syndrome, *MHNW* metabolically healthy normal weight, *MHO* metabolically healthy, but obese, *MANW* metabolically abnormal, but normal weight, *MAO* metabolically abnormal obese

The percentage of metabolically abnormal men among the obese subjects was 51.2 %, whereas that among men with normal weight was 16.5 % (Fig. 3). Normal weight subjects showed no associations between increased mean CIMT value, carotid plaque, carotid atherosclerosis, and MetS. Among the obese subjects, aORs for increased CIMT, the presences of carotid plaque and atherosclerosis in metabolically abnormal men were 1.80 (95 % CI 1.02–3.17; p = 0.041), 1.66 (95 % CI 1.02–2.67; p = 0.039) and 1.69 (95 % CI 1.07–2.65; p = 0.023), respectively, compared to metabolically healthy men (Table 4).

**Table 4:** Association between carotid atherosclerosis and metabolic syndrome according to obesity

	Increased CIMT	p value	Plaque	p value	Carotid atherosclerosis	p value
MHNW	1		1	–	1	–
MANW	0.74 (0.34–1.59)	0.443	0.59 (0.31–1.16)	0.127	0.70 (0.33–1.51)	0.364
MHO	1		1	–	1	–
MAO	*1.80* (*1.02*–*3.17*)	*0.041*	*1.66* (*1.02*–*2.67*)	*0.039*	*1.69* (*1.07*–*2.65*)	*0.023*

Values are expressed as odds ratio (95 % confidence interval). Data were analysed with adjusting for age, smoking, alcohol consumption, physical activity, total cholesterol, LDL-C, HOMA-IR index and eGFR levels. Results in italics indicate statistical significance at the 0.05 level

*CIMT* carotid intima-media thickness, *MANW* metabolically abnormal, but normal weight, *MAO* metabolically abnormal obese, *MHO* metabolically healthy, but obese, *MHNW* metabolically healthy normal weight

## Discussion

We investigated the associations between CIMT, carotid plaque, carotid atherosclerosis and subtypes of obesity in Korean men. Mean CIMT in the MAO subtype was significantly higher than that in MHNW controls. The presence of carotid plaque was positively associated with MAO subtype, but not with MHO or MANW, compared to MHNW controls. Only the MAO subtype showed a positive association with the presence of carotid atherosclerosis.

The occurrence of clinical CVD/stroke events from the presence of the associated risk factors usually need for a long-term period [24]. The confirmation of subclinical atherosclerosis, such as abnormalities in left ventricular structure and function [25], coronary calcification [15], and increased CIMT [16] could better identify the influence of cardiovascular risk factors, such as obesity and metabolic abnormalities alone or in combination, within a shorter period than the estimation of overt CVD/stroke events [24, 26]. In this regard, the measurement of subclinical carotid atherosclerosis is an appropriate predictor for evaluating the risks of CVD/stroke before the occurrence of such events [11, 12, 27].

We measured not only mean CIMT, but also the presence and severity of carotid plaque for assessment of carotid atherosclerosis. As reported in several previous studies, the presence of carotid plaque was a stronger predictor of CVD/stroke events than the measurement of CIMT [14, 28]. Furthermore, carotid plaque provided a superior negative predictive value and likelihood ratio of a negative test than CIMT as well as coronary calcium score and C-reactive protein, which are other independent predictors of CVD events [29]. In this study, the presence of carotid plaque was positively associated with MAO subtype, but not with MHO and MANW subtypes. Interestingly, however, the presence of high-risk or vulnerable carotid plaque was not associated with any subtype of obesity, but moderate-risk plaque showed a positive relationship with MAO subtype. These results may have been due to the close correlation of the features of high-risk plaque with previous CVD/stroke events [10]; subjects with a history of previous CVD/stroke events were excluded from this analysis.

In the present study, the MHO subtype did not show risks of carotid atherosclerosis. However, these results must be interpreted with caution. Because the participants with the MHO subtype were younger than those with the MAO subtype, the MHO subtype may transition from obese status without metabolic abnormalities to obese status with metabolic abnormalities for a long time, and the clinical outcomes including vascular change may manifest after long-term follow-up [5]. Indeed, in a previous prospective study, the subjects who maintained their baseline metabolic status after follow-up in MHO subtype showed similar developments of CVD compared to MHNW controls [5], suggesting that targeted intervention may be needed to maintain favourable metabolic health in younger MHO subtype Koreans.

Several studies reported that the MANW subtype show increased rates of CVD/stroke events than MHNW controls [6], whereas the results in other studies do not [5]. In fact, the phenotype of NAMW subtype could show lipodystrophy that is insufficient adipose tissue, especially subcutaneous depots, to carry out the normal buffering function of lipid flux and leads to the lipotoxicity which is an over-accumulation of triacylglycerol in abnormal sites such as skeletal muscle, liver and pancreas, and could be a causal factor of insulin resistance [30]. Additionally, MANW subtype showed higher concentrations of oxidized low-density lipoprotein and inflammatory cytokines such as tumor necrosis factor-α and interleukin-6, compared with MHNW control [31], suggesting that MANW subtype could relate more to the presence or the developments of CVD/stroke events. MANW subtype is not rare with a prevalence of 3–28 % depending on the definition of it and the ethnicity. In this study, the prevalence of MANW subtype in Korean men was 8.2 % overall and 16.5 % among those with normal weight. In particular, Asian populations have higher prevalence rates of MANW subtype than Caucasian populations [32, 33], and the ethnic differences may be attributable to higher visceral adipose tissue at the same BMI levels in Asians compared to Caucasians [34].

However, in this study, in spite of multiple metabolic abnormalities such as elevated fasting glucose, triglycerides, HOMA-IR index, and blood pressure, and low HDL-C in MANW subtype, there were no associations with carotid atherosclerosis compared to MHNW controls. Because many participants with well-controlled diabetes or hypertension were included in MANW subtype of this study and the proper management to maintain good medical condition might contribute to these results, the interpretation of the results needs to be made with caution. Further prospective trials are necessary to more certainly settle this issue in the MANW subtype of Korean men.

Not surprisingly but importantly, MAO subtype as the concomitant presence of obesity and MetS showed a positive association with carotid atherosclerosis in Korean men, consistent with previous reports [35]. In this regard, the consideration for the presence of metabolic abnormalities may help to discriminate the high-risk obese status among Korean obese men for the prediction and prevention of CVD/stroke.

The strength of the present study was that as indicators of subclinical atherosclerosis, not only CIMT, but also the presence and severity of carotid plaque were measured, and from these measurements, carotid atherosclerosis was identified, and the associations between subtypes of obesity and the various predictors of CVD/stroke events were examined. However, this study had several limitations. First, cross-sectional analysis cannot be used to determine causal relationships. However, due to the lag time necessary for the transition from the presence of MetS or obesity to overt CVD/stroke events [24], the cross-sectional design would be more suitable for investigating the associations of subclinical atherosclerosis with the subtypes of obesity, rather than for evaluating the occurrence of overt CVD/stroke events according to the subtypes of obesity. Second, the concept of “overweight (23.0 ≤ BMI < 25.0 kg/m^2^)” from the definition of “normal weight (BMI < 25.0 kg/m^2^)” was not distinguished in this study. Third, we only included Korean men aged 19–80 years of age, therefore additional studies are needed to examine the associations of carotid atherosclerosis with obesity subtypes in Korean women.

## Conclusions

In Korean men, mean CIMT in the MAO subtype of obesity was higher than that in MHNW controls. Only the MAO subtype showed positive associations with the presence of carotid plaque and carotid atherosclerosis, whereas MHO and MANW subtypes did not, compared to MHNW controls. Additional prospective studies are needed to evaluate preclinical carotid atherosclerosis according to the subtypes of obesity to establish effective strategies for preventing CVD/stroke events and predicting the occurrence of CVD/stroke events according to obesity and metabolic abnormalities.

## Abbreviations

aOR: adjusted odds ratio
BMI: body mass index
CVD: cardiovascular disease
CIMT: carotid intima-media thickness
CI: confidence interval
eGFR: estimated glomerular filtration rate
HDL-C: high-density lipoprotein cholesterol
HOMA-IR: homeostasis model assessment insulin resistance
LDL-C: low-density lipoprotein cholesterol
MANW: metabolically abnormal, but normal weight
MAO: metabolically abnormal obese
MHNW: metabolically healthy normal weight
MHO: metabolically healthy, but obese
MetS: metabolic syndrome

## References

[1] Ng M, Fleming T, Robinson M, Thomson B, Graetz N, Margono C (2014). Global, regional, and national prevalence of overweight and obesity in children and adults during 1980–2013: a systematic analysis for the Global Burden of Disease Study 2013. Lancet.

[2] Chapman MJ, Sposito AC (2008). Hypertension and dyslipidaemia in obesity and insulin resistance: pathophysiology, impact on atherosclerotic disease and pharmacotherapy. Pharmacol Ther.

[3] Ruderman NB, Schneider SH, Berchtold P (1981). The, “metabolically-obese,” normal-weight individual. Am J Clin Nutr.

[4] Karelis AD, St-Pierre DH, Conus F, Rabasa-Lhoret R, Poehlman ET (2004). Metabolic and body composition factors in subgroups of obesity: what do we know?. J Clin Endocrinol Metab.

[5] Appleton SL, Seaborn CJ, Visvanathan R, Hill CL, Gill TK, Taylor AW (2013). Diabetes and cardiovascular disease outcomes in the metabolically healthy obese phenotype: a cohort study. Diabetes Care.

[6] Hamer M, Stamatakis E (2012). Metabolically healthy obesity and risk of all-cause and cardiovascular disease mortality. J Clin Endocrinol Metab.

[7] Kwon BJ, Kim DW, Her SH, Kim DB, Jang SW, Cho EJ (2013). Metabolically obese status with normal weight is associated with both the prevalence and severity of angiographic coronary artery disease. Metabolism.

[8] Choi KM, Cho HJ, Choi HY, Yang SJ, Yoo HJ, Seo JA (2013). Higher mortality in metabolically obese normal-weight people than in metabolically healthy obese subjects in elderly Koreans. Clin Endocrinol (Oxf).

[9] Bots ML (2006). Carotid intima-media thickness as a surrogate marker for cardiovascular disease in intervention studies. Curr Med Res Opin.

[10] Naqvi TZ, Lee MS (2014). Carotid intima-media thickness and plaque in cardiovascular risk assessment. JACC Cardiovasc Imaging.

[11] Johnsen SH, Mathiesen EB, Joakimsen O, Stensland E, Wilsgaard T, Løchen ML (2007). Carotid atherosclerosis is a stronger predictor of myocardial infarction in women than in men: a 6-year follow-up study of 6226 persons: the Tromso Study. Stroke.

[12] Grønholdt ML, Nordestgaard BG, Schroeder TV, Vorstrup S, Sillesen H (2001). Ultrasonic echolucent carotid plaques predict future strokes. Circulation.

[13] Belcaro G, Nicolaides AN, Ramaswami G, Cesarone MR, De Sanctis M, Incandela L (2001). Carotid and femoral ultrasound morphology screening and cardiovascular events in low risk subjects: a 10-year follow-up study (the CAFES-CAVE study(1)). Atherosclerosis.

[14] Inaba Y, Chen JA, Bergmann SR (2012). Carotid plaque, compared with carotid intima-media thickness, more accurately predicts coronary artery disease events: a meta-analysis. Atherosclerosis.

[15] Khan UI, Wang D, Thurston RC, Sowers M, Sutton-Tyrrell K, Matthews KA (2011). Burden of subclinical cardiovascular disease in “metabolically benign” and “at-risk” overweight and obese women: the Study of Women’s Health Across the Nation (SWAN). Atherosclerosis.

[16] Yoo HJ, Hwang SY, Hong HC, Choi HY, Seo JA, Kim SG (2014). Association of metabolically abnormal but normal weight (MANW) and metabolically healthy but obese (MHO) individuals with arterial stiffness and carotid atherosclerosis. Atherosclerosis.

[17] Laing ST, Smulevitz B, Vatcheva KP, Rahbar MH, Reininger B, McPherson DD (2015). Subclinical atherosclerosis and obesity phenotypes among Mexican Americans. J Am Heart Assoc.

[18] Wen CP, David Cheng TY, Tsai SP, Chan HT, Hsu HL, Hsu CC (2009). Are Asians at greater mortality risks for being overweight than Caucasians? Redefining obesity for Asians. Public Health Nutr.

[19] Grundy SM, Cleeman JI, Daniels SR, Donato KA, Eckel RH, Franklin BA (2005). Diagnosis and management of the metabolic syndrome: an American Heart Association/National Heart, Lung, and Blood Institute scientific statement. Circulation.

[20] Lee SY, Park HS, Kim DJ, Han JH, Kim SM, Cho GJ (2007). Appropriate waist circumference cutoff points for central obesity in Korean adults. Diabetes Res Clin Pract.

[21] Mancia G, Fagard R, Narkiewicz K, Redon J, Zanchetti A, Böhm M (2013). 2013 ESH/ESC guidelines for the management of arterial hypertension: the Task Force for the Management of Arterial Hypertension of the European Society of Hypertension (ESH) and of the European Society of Cardiology (ESC). Eur Heart J.

[22] Matthews DR, Hosker JP, Rudenski AS, Naylor BA, Treacher DF, Turner RC (1985). Homeostasis model assessment: insulin resistance and beta-cell function from fasting plasma glucose and insulin concentrations in man. Diabetologia.

[23] Levey AS, Coresh J, Greene T, Marsh J, Stevens LA, Kusek JW (2007). Expressing the modification of diet in renal disease study equation for estimating glomerular filtration rate with standardized serum creatinine values. Clin Chem.

[24] Arnlöv J, Ingelsson E, Sundström J, Lind L (2010). Impact of body mass index and the metabolic syndrome on the risk of cardiovascular disease and death in middle-aged men. Circulation.

[25] Park J, Kim SH, Cho GY, Baik I, Kim NH, Lim HE (2011). Obesity phenotype and cardiovascular changes. J Hypertens.

[26] Kramer CK, Zinman B, Retnakaran R (2013). Are metabolically healthy overweight and obesity benign conditions?: a systematic review and meta-analysis. Ann Intern Med.

[27] Den Ruijter HM, Peters SA, Anderson TJ, Britton AR, Dekker JM, Eijkemans MJ (2012). Common carotid intima-media thickness measurements in cardiovascular risk prediction: a meta-analysis. JAMA.

[28] Plichart M, Celermajer DS, Zureik M, Helmer C, Jouven X, Ritchie K (2011). Carotid intima-media thickness in plaque-free site, carotid plaques and coronary heart disease risk prediction in older adults. The Three-City Study. Atherosclerosis.

[29] Brook RD, Bard RL, Patel S, Rubenfire M, Clarke NS, Kazerooni EA (2006). A negative carotid plaque area test is superior to other noninvasive atherosclerosis studies for reducing the likelihood of having underlying significant coronary artery disease. Arterioscler Thromb Vasc Biol.

[30] Frayn KN (2002). Adipose tissue as a buffer for daily lipid flux. Diabetologia.

[31] Hyun YJ, Koh SJ, Chae JS, Kim JY, Kim OY, Lim HH (2008). Atherogenecity of LDL and unfavorable adipokine profile in metabolically obese, normal-weight woman. Obesity (Silver Spring).

[32] Kagawa M, Binns CB, Hills AP (2007). Body composition and anthropometry in Japanese and Australian Caucasian males and Japanese females. Asia Pac J Clin Nutr.

[33] Lee SH, Ha HS, Park YJ, Lee JH, Yim HW, Yoon KH (2011). Identifying metabolically obese but normal-weight (MONW) individuals in a nondiabetic Korean population: the Chungju Metabolic disease Cohort (CMC) study. Clin Endocrinol (Oxf).

[34] Katsuki A, Sumida Y, Urakawa H, Gabazza EC, Murashima S, Maruyama N (2003). Increased visceral fat and serum levels of triglyceride are associated with insulin resistance in Japanese metabolically obese, normal weight subjects with normal glucose tolerance. Diabetes Care.

[35] Fan J, Song Y, Chen Y, Hui R, Zhang W (2013). Combined effect of obesity and cardio-metabolic abnormality on the risk of cardiovascular disease: a meta-analysis of prospective cohort studies. Int J Cardiol.

